# Supplementary material for: Evolution of the germline mutation rate across vertebrates
Source: Nature. 2023 Mar 1;615(7951):285–91. doi: 10.1038/s41586-023-05752-y (PMC9995274; doi:10.1038/s41586-023-05752-y)
Supplement: Supplementary file 1 — This file contains the Supplementary Figs. 1–8. [file 41586_2023_5752_MOESM1_ESM.pdf]

nature portfolio

https://doi.org/10.1038/s41586-023-05752-y

Supplementary information

Evolution of the germline mutation rate across vertebrates

In the format provided by the
authors and unedited

Supplementary figures

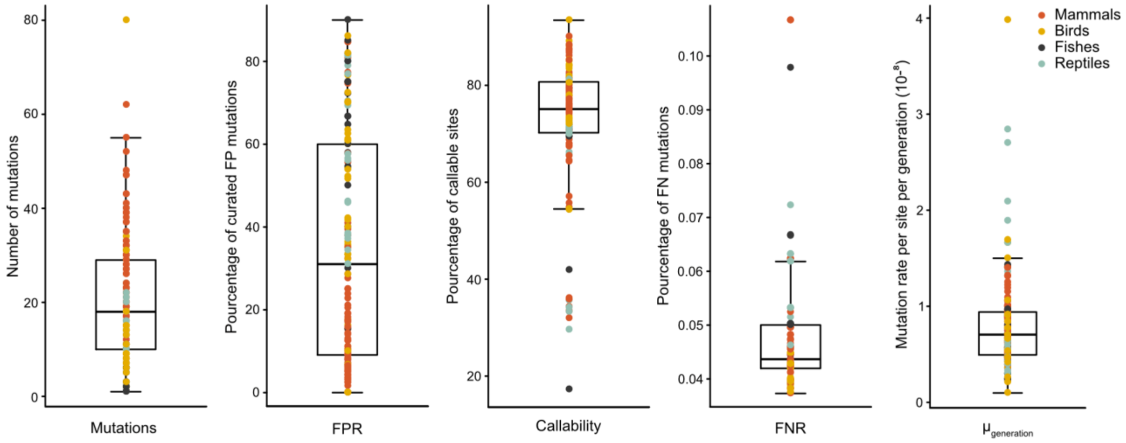

Supplementary Fig. 1 – Box plots of the various parameters used to estimate the germline mutation rate per site per generation for each of the 151 trios. From left to right: the number of DNMs detected, the percentage of false-positive calls among the candidate DNMs (i.e. the false-positive rate), the callable genome as a percentage of the whole genome, the false-negative rate (percentage), and finally, the per generation germline mutation rate. The boxplots represent the median, the interquartile range, and the maximum and minimum excluding outliers.

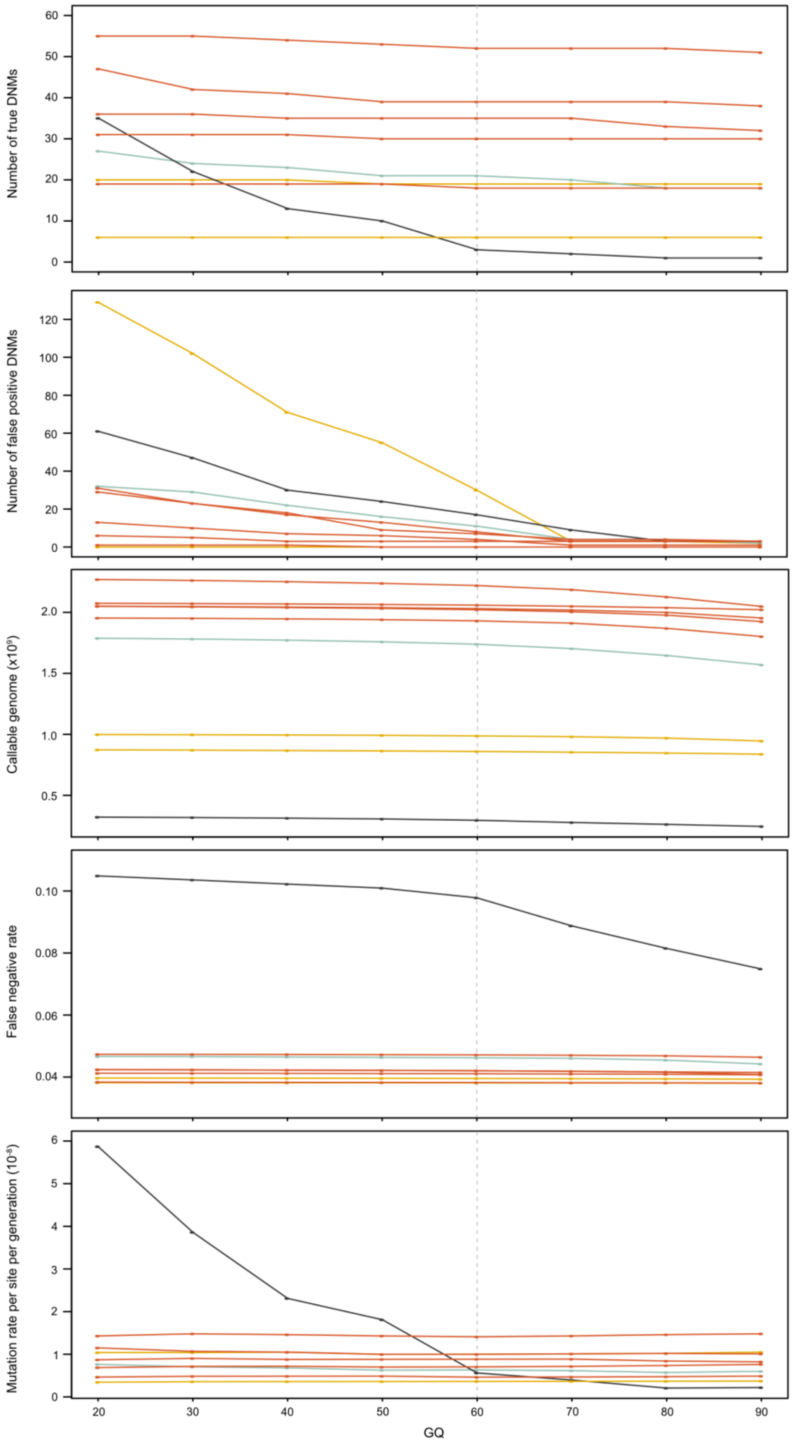

Supplementary Fig. 2 – Effect of the GQ filter. We tested the effect of varying the GQ filter using a representative subset of nine species (five mammals, two birds, one fish and one reptile). The number of true DNMs and the callable genome were consistent for most species (except for the fish), while the number of false positives decreased while GQ increased, and the false-negative rate also decreased for the fish species when GQ increased. The final rates that we obtained were reasonably robust to GQ variation because the denominators and numerators decreased similarly. However, the inferred mutation rate of the fish species decreased from lower to higher GQ and then stabilized above GQ = 60.

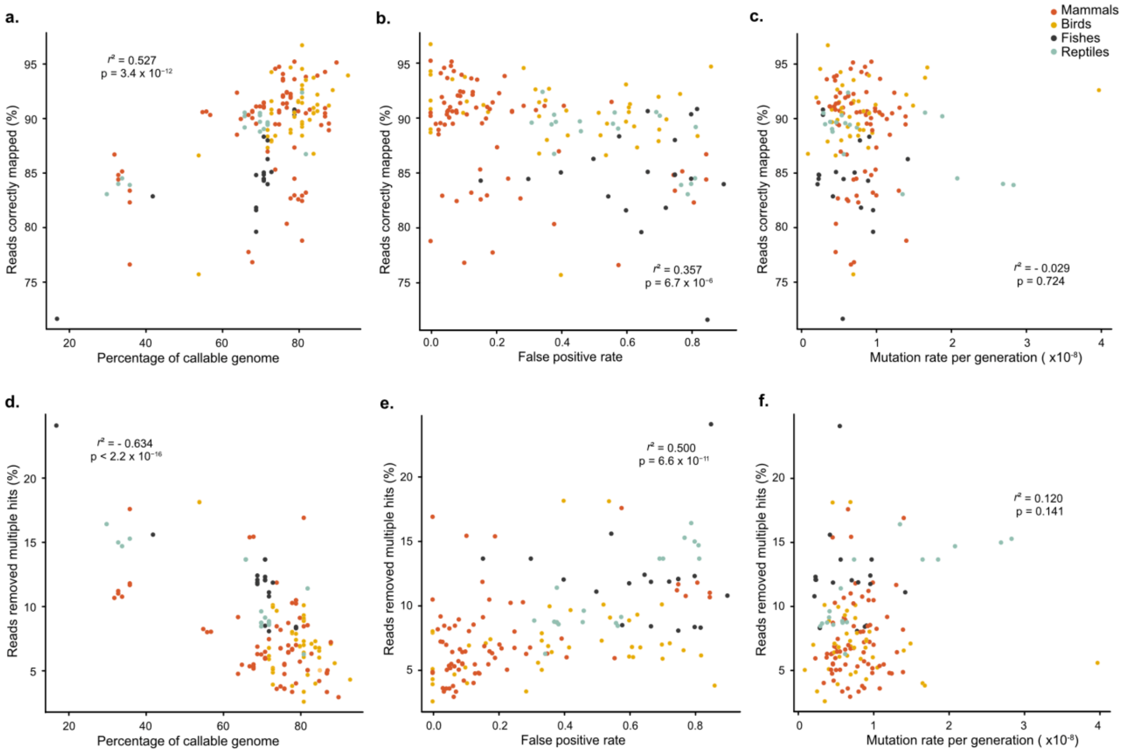

Supplementary Fig. 3 – The effect of mapping quality on the estimated mutation rates using phylogenetic regression (PGLS). a. A high proportion of reads that mapped correctly to the reference genome (an indicator of good mapping quality) corresponds to a high percentage of genome considered callable (PGLS: adjusted $r^2 = 0.53$, $p = 3.4 \times 10^{-12}$) and **b.** a lower false-positive rate (PGLS: adjusted $r^2 = 0.36$, $p = 6.7 \times 10^{-6}$). **c.** However, this does not affect the final mutation rate estimates (PGLS: adjusted $r^2 = -0.03$, $p = 0.72$). **d.** Conversely, a high percentage of reads removed due to multiple mapping (an indicator of poor mapping quality) corresponds to a lower callable genome (PGLS: adjusted $r^2 = -0.63$, $p < 2.2 \times 10^{-16}$) and **e.** a higher percentage of false-positive calls (PGLS: adjusted $r^2 = 0.5$, $p = 6.6 \times 10^{-11}$). **f.** However, here again, the final mutation rate estimates are not affected (PGLS: adjusted $r^2 = 0.12$, $p = 0.14$).

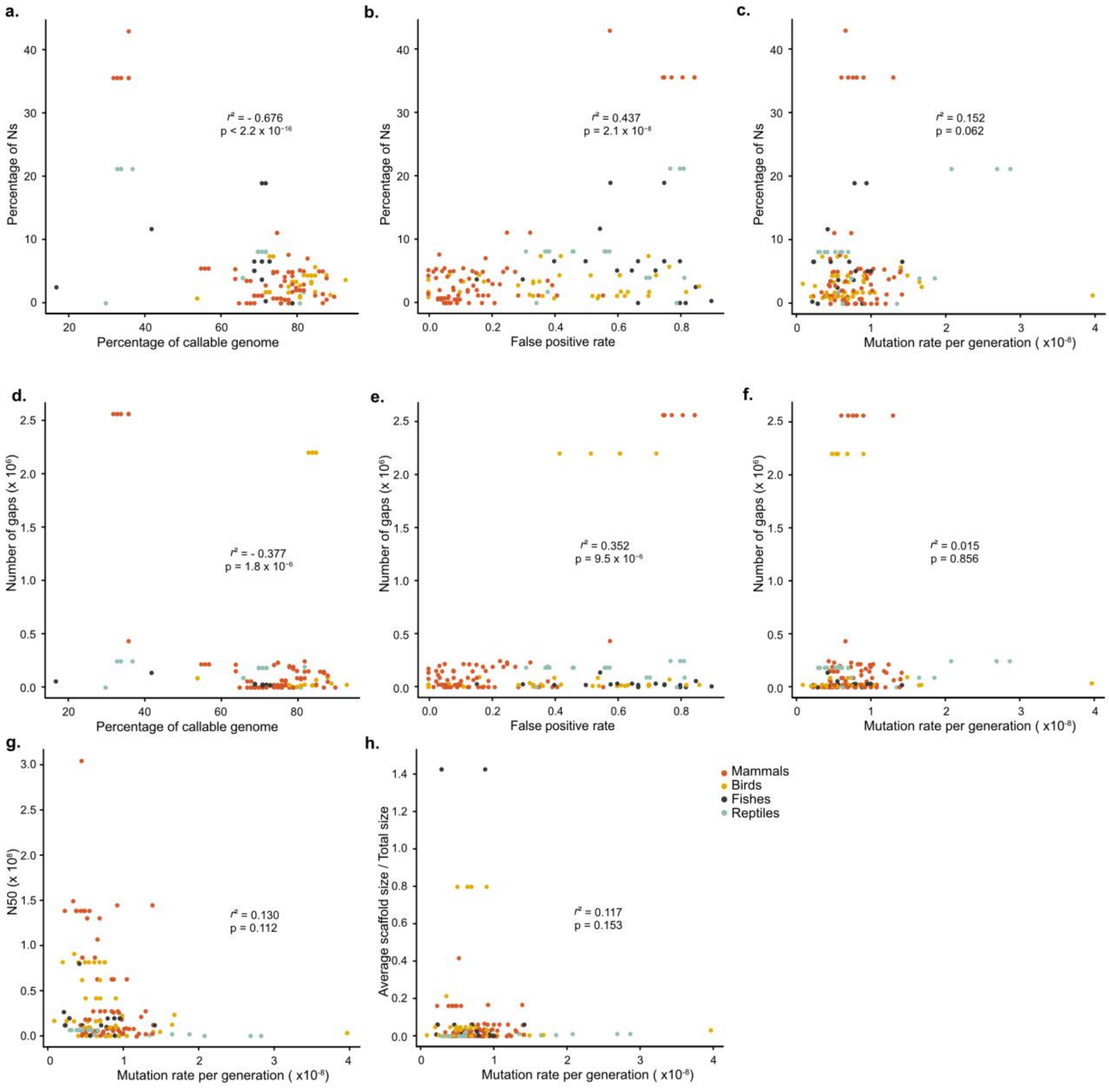

Supplementary Fig. 4 – The effect of reference genome quality on the estimated mutation rates using phylogenetic regression (PGLS). a. The proportion of Ns in the reference genome is associated with the percentage of callable genome (a higher proportion of Ns leads to lower callable genomes) (PGLS: adjusted r^2 = -0.68, p < 2.2 × 10^{-16}) and **b.** the number of false positives (a higher proportion of Ns leads to more false-positive calls) (PGLS: adjusted r^2 = 0.44, p = 2.1 × 10^{-8}), **c.** However, the proportion of Ns does not affect the final mutation rate estimates (PGLS: adjusted r^2 = 0.15, p = 0.06). **d.** Similar observations were performed on the number of gaps; we find a negative correlation between the number of gaps and callable genome (PGLS: adjusted r^2 = -0.38, p = 1.8 × 10^{-6}), **e.** a positive correlation with the false-positive rate (PGLS: adjusted r^2 = 0.35, p = 9.5 × 10^{-6}), and **f.** no effect on the final mutation rate estimates (PGLS: adjusted r^2 = 0.02, p = 0.86). **g**. The N50 and **h**. the average scaffold size also does not affect the mutation rate per generation (PGLS: adjusted r^2 = 0.13, p = 0.11 and adjusted r^2 = 0.12, p = 0.15).

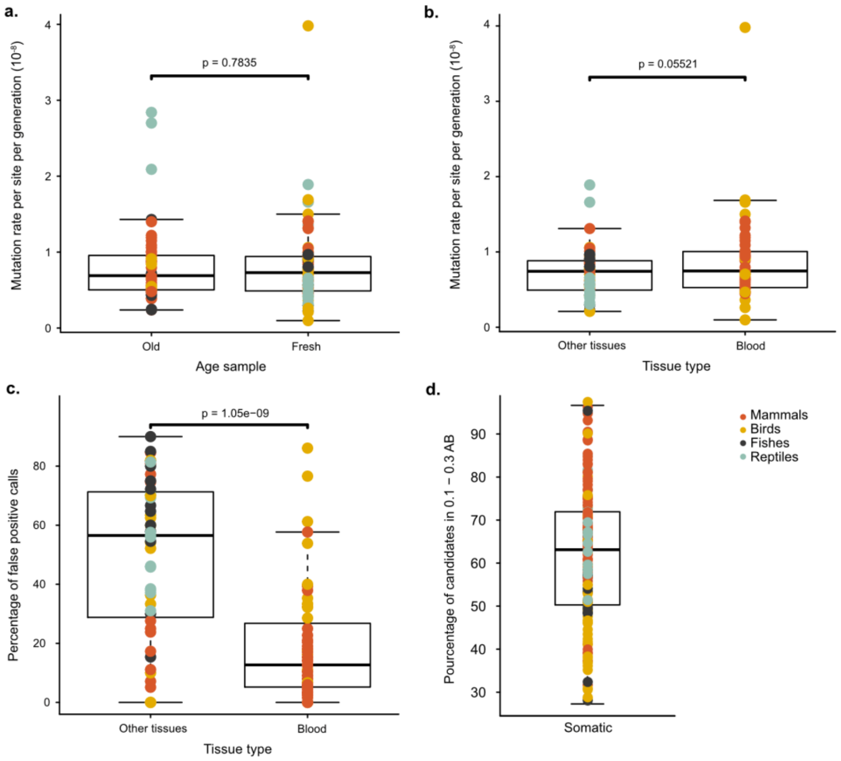

Supplementary Fig. 5 – The effect of tissue type on the estimated mutation rates, using phylogenetic regression (PGLS). a. We find no significant effect of the age of the tissue (fresh or stored) on the mutation rate. **b.** Similarly, mutation rate estimates do not vary significantly by tissue type. **c.** However, tissue type tends to affect the percentage of false-positive calls, with fewer false positives obtained for blood than for other tissues. **d.** We examined the percentage of candidate mutations with an allelic balance of between 10 % and 30 % of the reads which could correspond to somatic mutations and found appreciable variation among species. In this analysis, we used a total of 151 trios. The boxplots represent the median, the interquartile range, and the maximum and minimum excluding outliers.

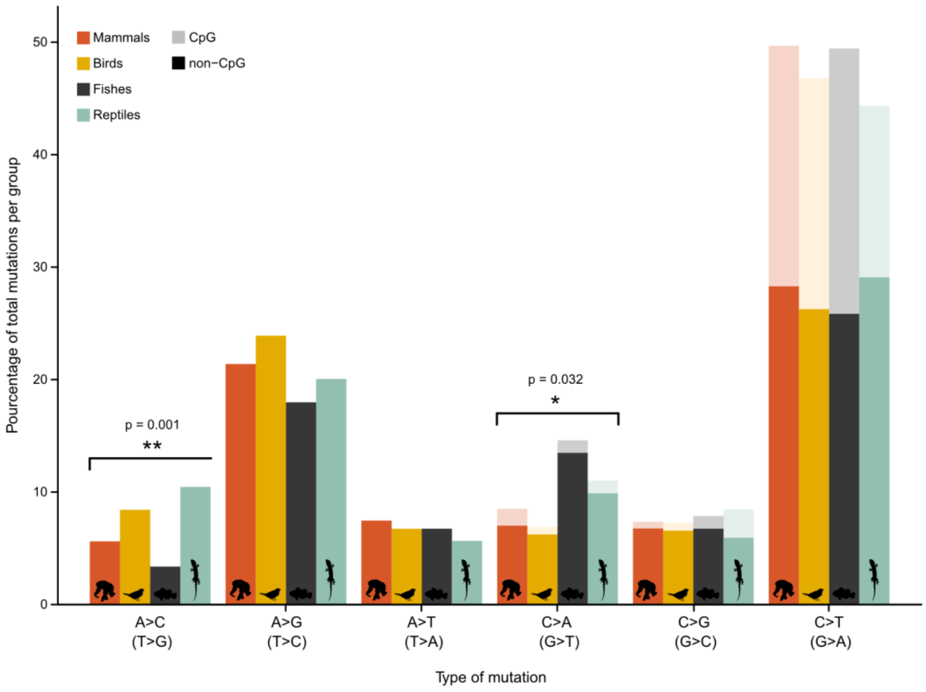

Supplementary Fig. 6 – Germline mutation spectra. Spectra of mutations show significant differences among mammals, birds, fishes, and reptiles for A > C mutations (χ^2 one-sided = 16.2, df = 3, p = 0.001) and C > A mutations (χ^2 one-sided = 8.8, df = 3, p = 0.032). Differences are not significant for the other types of mutation. The percentages of all mutations located in CpG sites (indicated by the translucent bars) also does not differ significantly among the taxonomic groups (χ^2 one-sided = 4.3, df = 3, p = 0.23). All silhouettes are from PhyloPic (http://phylopic.org), except one of the silhouettes of *Pan troglodytes*, which was created by T. M. Keesey (vectorization) and T. Hisgett (photography) and is available under a CC-BY 3.0 licence (https://creativecommons.org/licenses/by/3.0).

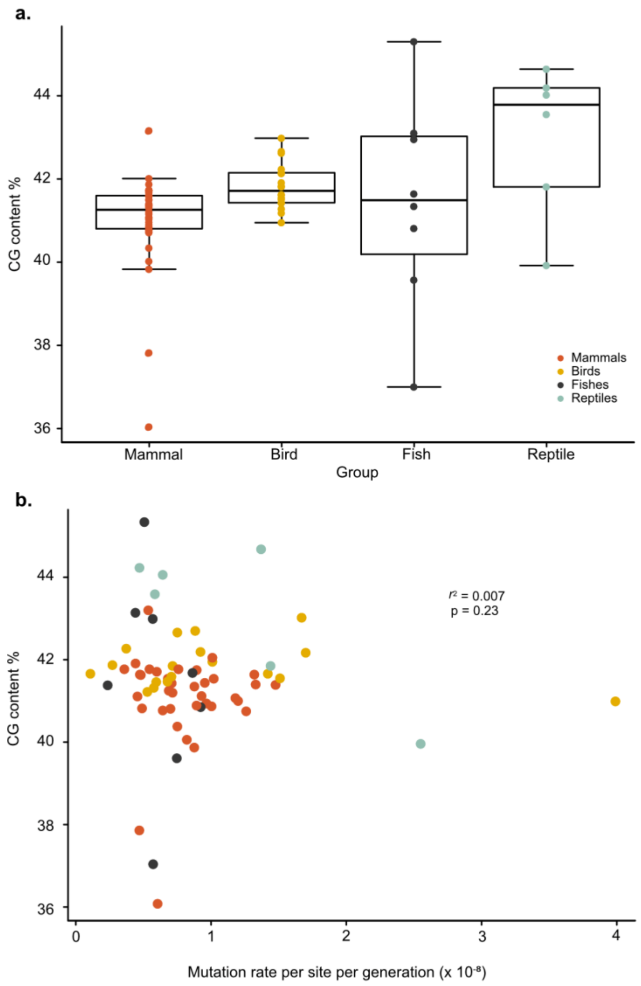

Supplementary Fig. 7 – Variation in CG content among classes of vertebrates. **a.** Box plots of CG content in the reference genomes of the 68 vertebrate species. The boxplots represent the median, the interquartile range, and the maximum and minimum excluding outliers**. b.** Overall, GC content is not significantly associated with the observed mutation rate per generation (PGLS: adjusted r^2 = 0.007, p = 0.23).

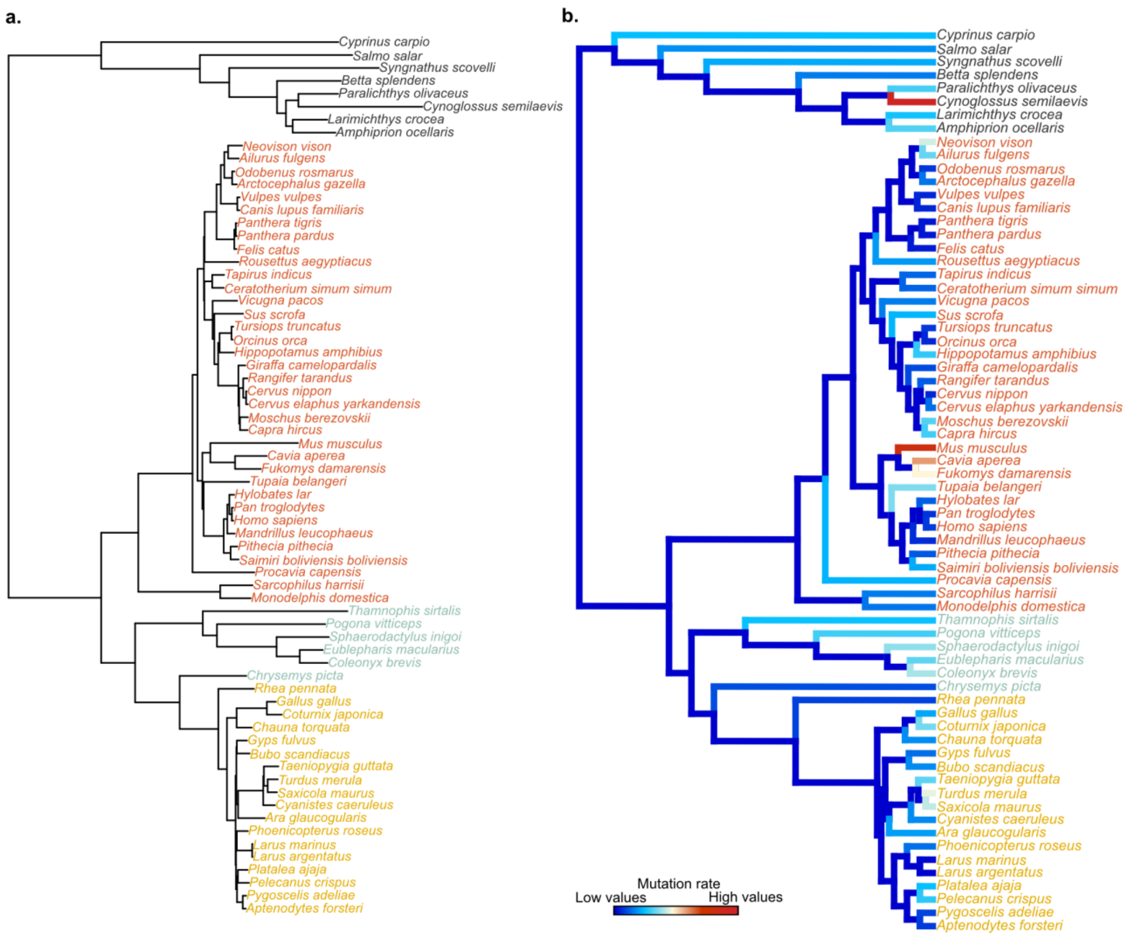

Supplementary Fig. 8 – Phylogenetic trees based on ultraconserved elements (UCEs). **a.** The branch lengths of the UCE tree show patterns of substitution accumulation for the 68 species. **b**. The UCE tree was calibrated at 14 nodes to estimate the substitution rates of the terminal branches, which vary from low substitution rates in blue to high substitution rates in red.
