# Supplementary material for: Evolution of the germline mutation rate across vertebrates
Source: Nature. 2023 Mar 1;615(7951):285–91. doi: 10.1038/s41586-023-05752-y (PMC9995274; doi:10.1038/s41586-023-05752-y)
Supplement: Supplementary file 2 — Reporting Summary [file 41586_2023_5752_MOESM2_ESM.pdf]

nature portfolio

Corresponding author(s): Lucie A. Begeron and Guojie Zhang

Last updated by author(s): Jan 2, 2023

Reporting Summary

Nature Portfolio wishes to improve the reproducibility of the work that we publish. This form provides structure for consistency and transparency in reporting. For further information on Nature Portfolio policies, see our Editorial Policies and the Editorial Policy Checklist.

Statistics

For all statistical analyses, confirm that the following items are present in the figure legend, table legend, main text, or Methods section.

n/a	Confirmed	
☐	☒	The exact sample size (*n*) for each experimental group/condition, given as a discrete number and unit of measurement
☐	☒	A statement on whether measurements were taken from distinct samples or whether the same sample was measured repeatedly
☐	☒	The statistical test(s) used AND whether they are one- or two-sided<br>*Only common tests should be described solely by name; describe more complex techniques in the Methods section.*
☐	☒	A description of all covariates tested
☐	☒	A description of any assumptions or corrections, such as tests of normality and adjustment for multiple comparisons
☐	☒	A full description of the statistical parameters including central tendency (e.g. means) or other basic estimates (e.g. regression coefficient) AND variation (e.g. standard deviation) or associated estimates of uncertainty (e.g. confidence intervals)
☐	☒	For null hypothesis testing, the test statistic (e.g. *F*, *t*, *r*) with confidence intervals, effect sizes, degrees of freedom and *P* value noted<br>*Give P values as exact values whenever suitable.*
☐	☒	For Bayesian analysis, information on the choice of priors and Markov chain Monte Carlo settings
☐	☒	For hierarchical and complex designs, identification of the appropriate level for tests and full reporting of outcomes
☐	☒	Estimates of effect sizes (e.g. Cohen's *d*, Pearson's *r*), indicating how they were calculated

Our web collection on statistics for biologists contains articles on many of the points above.

Software and code

Policy information about availability of computer code

Data collection	No software was used for data collection
Data analysis	The software used were: samtools 1.2 and 0.1.18, bcftools 1.2 and 1.9, bwa 0.7.15, Picard MarkDuplicates 2.7.1, R 3.5.1 and 4.0.2, IQTREE 2.0.3, Phyluce, ANGSD 0.920, soapnuke 1.5.6, python 3.7.3, java 1.8.0_222, gatk 4.0.7.0.<br>The entire pipeline used is available at https://github.com/lucieabergeron/vertebrate_rate.

For manuscripts utilizing custom algorithms or software that are central to the research but not yet described in published literature, software must be made available to editors and reviewers. We strongly encourage code deposition in a community repository (e.g. GitHub). See the Nature Portfolio guidelines for submitting code & software for further information.

Data

Policy information about availability of data

All manuscripts must include a data availability statement. This statement should provide the following information, where applicable:

- Accession codes, unique identifiers, or web links for publicly available datasets
- A description of any restrictions on data availability
- For clinical datasets or third party data, please ensure that the statement adheres to our policy

Whole-genome sequences of all species except humans are accessible in NCBI (National Center for Biotechnology Information) under the BioProject: PRJNA767781

(https://www.ncbi.nlm.nih.gov/bioproject/767781). The human sequences are available upon request. The alignments for the UCE tree are available on Figshare (https://doi.org/10.6084/m9.figshare.19221693.v1).

Human research participants

Policy information about studies involving human research participants and Sex and Gender in Research.

Reporting on sex and gender	Not applicable
Population characteristics	Not applicable
Recruitment	Not applicable
Ethics oversight	Not applicable

Note that full information on the approval of the study protocol must also be provided in the manuscript.

Field-specific reporting

Please select the one below that is the best fit for your research. If you are not sure, read the appropriate sections before making your selection.

☐ Life sciences ☐ Behavioural & social sciences ☒ Ecological, evolutionary & environmental sciences

For a reference copy of the document with all sections, see nature.com/documents/nr-reporting-summary-flat.pdf

Ecological, evolutionary & environmental sciences study design

All studies must disclose on these points even when the disclosure is negative.

Study description	This study estimates the germline mutation rates for 68 species of vertebrates using whole genome comparison of pedigrees.
Research sample	We analyzed 151 trios for 68 species of vetebrates, including birds, reptiles, fishes and mammals.
Sampling strategy	We collected trios (mother, father, offspring) for species with reference genome available. Our sample size for each species was limited by the pedigree available.
Data collection	Lucie A. Bergeron collected all the data, with the help of collaborators from zoo, research centers and farm.
Timing and spatial scale	Data were collected between October 2017 and December 2018.
Data exclusions	After data analysis, some of the samples were not as related as stated by the samples providers, therefore, they were excluded.
Reproducibility	All sequences and scripts are provided for reproducibility of our results.
Randomization	This is not relevant to our study, randomization was not possible as the sample size was small per species.
Blinding	Blinding was not relevant to our study as there were no expected data most of the rates were never estimated before from pedigrees.

Did the study involve field work? ☐ Yes ☒ No

Reporting for specific materials, systems and methods

We require information from authors about some types of materials, experimental systems and methods used in many studies. Here, indicate whether each material, system or method listed is relevant to your study. If you are not sure if a list item applies to your research, read the appropriate section before selecting a response.

Materials & experimental systems

n/a	Involved in the study
☒	☐ Antibodies
☒	☐ Eukaryotic cell lines
☒	☐ Palaeontology and archaeology
☒	☐ Animals and other organisms
☒	☐ Clinical data
☒	☐ Dual use research of concern

Methods

n/a	Involved in the study
☒	☐ ChIP-seq
☒	☐ Flow cytometry
☒	☐ MRI-based neuroimaging
