# Supplementary material for: Evolution of the germline mutation rate across vertebrates
Source: Nature. 2023 Mar 1;615(7951):285–91. doi: 10.1038/s41586-023-05752-y (PMC9995274; doi:10.1038/s41586-023-05752-y)
Supplement: Supplementary file 4 — This file contains detailed information on the model used to estimate germline mutation rate per generation at generation time. [file 41586_2023_5752_MOESM4_ESM.pdf]

Calculating Poisson models of vertebrate mutations using trios with recorded parental ages

Contents

1 Model of the effect of parental age **1**

2 Differences between species **2**

3 Correcting observed generation times. **4**

4 Validating model choice **7**

4.1 Checking assumptions on parameters 7

4.2 Model selection 8

This supplement outlines our modelling of the relationship between the number of mutations per generation and the ages and relative contributions of the parents. We have modeled the relationship between between the number of mutations, the number of callable bases and the age of the parents using Bayesian Poisson models. For this analysis we have only used trios with recorded parental ages (105 out of 151 trios from 56 out of 68 species) and we have furthermore excluded the outlier "Darwin's Rhea" leaving 104 trios from 55 species. The models are implemented using the probabilistic programming language STAN.

1 Model of the effect of parental age

To account for the effect of parental age we create a Poisson model describing the effect of parental age on the number of mutations. For most species the age of the father is most important but for some the age of the mother is more important. To take this into account we use a weighted average of the age of the parents using our estimate of the fraction of mutations originating from the father for the species.

$$\text{age\_mix}_i = (p_{s_i} \times \text{age\_father}_i + (1 - p_{s_i}) \times \text{age\_mother}_i) \tag{1}$$

Where p_s is the paternal fraction observed using read-backed phasing in species s and s_i is the specie of trio i. And age_father$_i$ and age_mother$_i$ are the ages when the child is born of the father and mother of the i'th trio. Model comparisons show that the model using this age_mix variable rather than just the age_father results in a slightly better model fit.

$$\begin{aligned} n_i &\sim \text{Poisson}(\lambda_i) \\ \lambda_i &= \text{denominator}_i \times (a + b \times \text{age\_mix}_i) \\ a &\sim \text{Exp}(1) \\ b &\sim \text{Exp}(1) \end{aligned} \tag{2}$$

Where n_i is the number of mutations in the i'th trio and

$$\text{denominator}_i = 2 \times \text{Callability} \times (1 - \text{FNR}_i) \tag{3}$$

is the denominator of the i'th trio. Using the exponential function as prior means that both the intercept and slope are required to be positive. But that is a fair assumption since it is impossible to remove mutations that have already occurred.

Table 1: Coefficients of model

coef	mean	sd	2.5%	97.5%
a	5.646e-09	2.12e-10	5.244e-09	6.065e-09
b	2.510e-10	2.20e-11	2.080e-10	2.960e-10

The model fits most of the trios well:

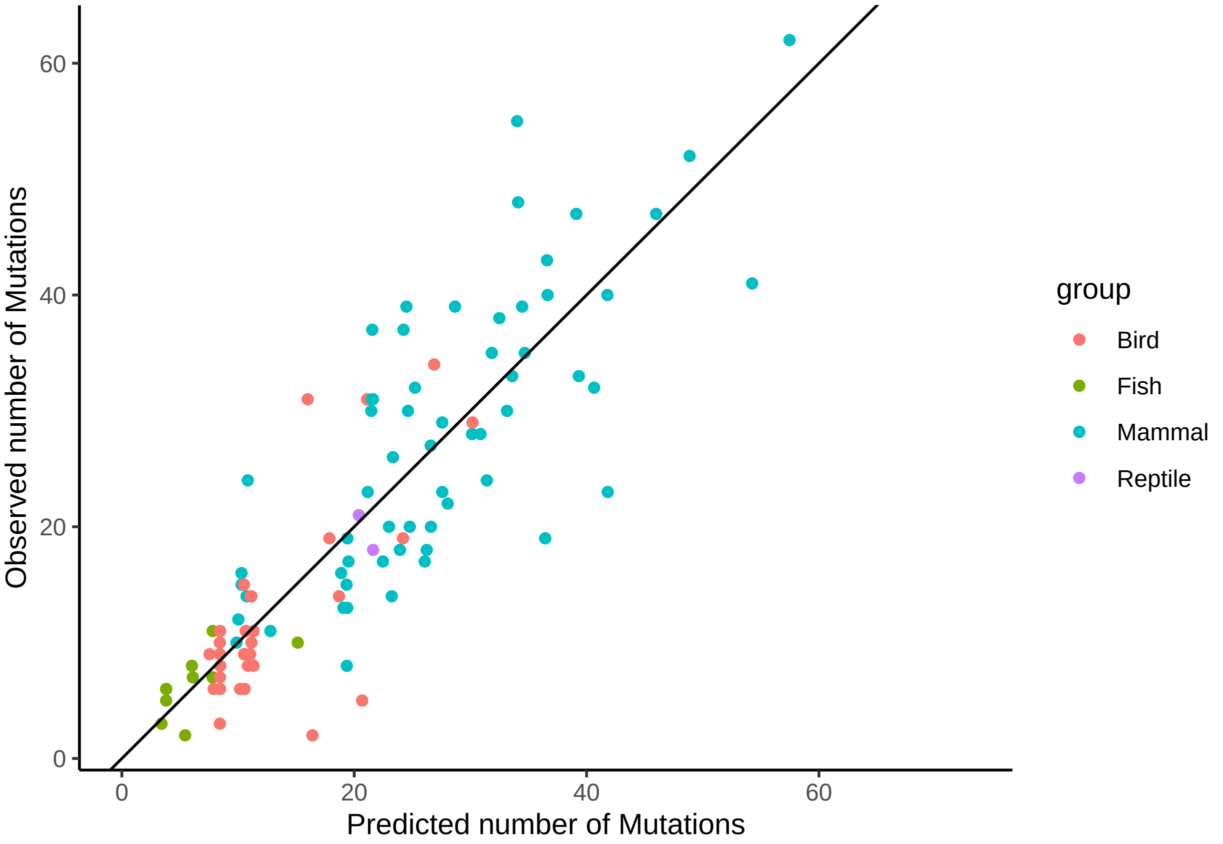

Figure 1: Observed number of mutations per trio compared to expected number given model.

2 Differences between species

To model deviations from the model and to see of which species tend to have higher (or lower) rate, compared to what is expected given this model, we created a model where we have a species specific scaling factor x for each species.

$$
\begin{aligned}
n_i &\sim \text{Poisson}(\lambda_i) \\
\lambda_i &= \text{denominator}_i \times (5.6457625 \times 10^{-9} + b \times \text{age\_mix}_i) \times x[s_i] \\
b &\sim \text{Exp}(1). \\
x[s_i] &\sim \text{LogNormal}(0, 1)
\end{aligned}
\tag{4}
$$

The table below show the fitted parameters for this model:

Table 2: Coefficients of model with species specific scaling factor

coef	mean	sd	2.5%	97.5%
b	2.720000e-10	1.020000e-10	9.800000e-11	4.960000e-10
x[Adelie penguin]	1.444097e+00	3.614766e-01	8.580928e-01	2.266674e+00
x[Alpaca]	1.408310e+00	2.596770e-01	9.492366e-01	1.966171e+00
x[Atlantic salmon]	6.840002e-01	2.077183e-01	3.453988e-01	1.151526e+00
x[Blue tits]	8.058930e-01	1.592474e-01	5.250719e-01	1.149767e+00
x[Blue-throated macaw]	1.053043e+00	2.856264e-01	5.930930e-01	1.705604e+00
x[Bolivian squirrel monkeys]	9.452403e-01	1.280769e-01	7.108411e-01	1.210543e+00
x[Bottlenosed dolphin]	7.894605e-01	2.031017e-01	4.618863e-01	1.249247e+00
x[Cervus nippon]	6.936969e-01	1.623248e-01	4.125788e-01	1.049632e+00
x[Chicken]	6.373426e-01	2.379764e-01	2.661194e-01	1.183010e+00
x[Chimpanzee]	1.190140e+00	2.729477e-01	7.357261e-01	1.809464e+00
x[Common blackbird]	9.459151e-01	1.634515e-01	6.593463e-01	1.294920e+00
x[Common carp]	9.271603e-01	4.525121e-01	2.797931e-01	2.002424e+00
x[Common clown fish]	1.136033e+00	2.653597e-01	6.800767e-01	1.718784e+00
x[Dalmatian pelican]	7.918099e-01	2.352272e-01	4.223335e-01	1.336477e+00
x[Dog]	1.400063e+00	1.338849e-01	1.152606e+00	1.675381e+00
x[Domestic cat]	6.231344e-01	1.568519e-01	3.546691e-01	9.661837e-01
x[Drill]	6.883456e-01	1.365750e-01	4.543672e-01	9.870250e-01
x[Emperor penguin]	9.664034e-01	2.941447e-01	5.238003e-01	1.671142e+00
x[Forest musk deer]	1.566293e+00	2.576342e-01	1.100640e+00	2.114711e+00
x[Giraffe]	1.336528e+00	2.459136e-01	9.065357e-01	1.880940e+00
x[Goat]	8.561079e-01	1.072921e-01	6.571300e-01	1.079530e+00
x[Gray short-tailed opossums]	7.694131e-01	1.549336e-01	4.952466e-01	1.100067e+00
x[Greater flamingo]	1.259747e+00	3.558050e-01	7.042057e-01	2.085878e+00
x[Griffon vulture]	2.994387e-01	1.226488e-01	1.181427e-01	5.876396e-01
x[Guinea pig]	1.454412e+00	1.558642e-01	1.167808e+00	1.772321e+00
x[Hippopotamus]	1.085055e+00	2.635088e-01	6.563862e-01	1.692923e+00
x[House mouse]	8.704997e-01	1.924332e-01	5.361170e-01	1.290164e+00
x[Human]	9.237382e-01	2.211509e-01	5.745084e-01	1.444894e+00
x[Japanese flounder]	1.209983e+00	3.102088e-01	6.840233e-01	1.894164e+00
x[Killer whale]	1.065634e+00	2.847242e-01	6.246625e-01	1.731155e+00
x[Lar gibbon]	8.987489e-01	1.877206e-01	5.757849e-01	1.305331e+00
x[Large yellow croaker]	5.112605e-01	2.637229e-01	1.434672e-01	1.151871e+00
x[Leopard]	1.084274e+00	2.189567e-01	7.067149e-01	1.567281e+00
x[Leopard gecko]	8.331656e-01	1.981976e-01	4.945238e-01	1.264345e+00
x[Malay tapir]	5.337944e-01	1.412775e-01	3.039514e-01	8.503981e-01
x[Neovison vison]	7.606309e-01	1.757024e-01	4.599266e-01	1.141895e+00
x[Pig]	7.493956e-01	7.537595e-02	6.081014e-01	9.029086e-01
x[Quails]	9.599350e-01	1.200284e-01	7.400984e-01	1.210762e+00

coef	mean	sd	2.5%	97.5%
x[Red deer]	8.652871e-01	2.676675e-01	4.295325e-01	1.479825e+00
x[Red fox]	7.690813e-01	1.802882e-01	4.562367e-01	1.156298e+00
x[Red panda]	1.490897e+00	2.484193e-01	1.057830e+00	2.031937e+00
x[Reindeer]	8.663102e-01	1.425412e-01	6.137054e-01	1.170031e+00
x[Rock hyrax]	1.318786e+00	1.611772e-01	1.021268e+00	1.652172e+00
x[Roseate spoonbill]	1.879223e+00	4.227312e-01	1.169364e+00	2.824934e+00
x[Siberian stonechat]	1.019481e+00	1.461774e-01	7.534687e-01	1.326739e+00
x[Snowy owl]	2.191650e-01	1.041417e-01	7.156749e-02	4.704268e-01
x[Southern screamer]	7.588476e-01	2.240331e-01	4.003374e-01	1.268789e+00
x[Southern White Rhinoceros]	9.524198e-01	2.169911e-01	5.925047e-01	1.438394e+00
x[Tasmanian devil]	9.254170e-01	1.789353e-01	6.108898e-01	1.317211e+00
x[Texas banded gecko]	1.024130e+00	2.195399e-01	6.385194e-01	1.490266e+00
x[Tiger]	1.040945e+00	2.001379e-01	6.916587e-01	1.476407e+00
x[Tongue sole]	1.449369e+00	3.463489e-01	8.511340e-01	2.206169e+00
x[Walrus]	1.025101e+00	2.722331e-01	5.989117e-01	1.654001e+00
x[White-faced saki]	1.034088e+00	1.801865e-01	7.252073e-01	1.428954e+00
x[Zebra finches]	9.695037e-01	2.420458e-01	5.575009e-01	1.499950e+00

For most species the 95% credible intervals (highest posterior density intervals) overlap 1. But we see a few species where the lower bound is higher than 1.

Table 3: Species with higher than expected mutation rate

Common name	mean_x	upper_x	lower_x
Roseate spoonbill	1.8792	2.8249	1.1694
Forest musk deer	1.5663	2.1147	1.1006
Red panda	1.4909	2.0319	1.0578
Guinea pig	1.4544	1.7723	1.1678
Dog	1.4001	1.6754	1.1526
Rock hyrax	1.3188	1.6522	1.0213

And we find a few species where the upper bound is lower than 1.

Table 4: Species with lower than expected mutation rate

Common name	mean_x	upper_x	lower_x
Snowy owl	0.2192	0.4704	0.0716
Griffon vulture	0.2994	0.5876	0.1181
Malay tapir	0.5338	0.8504	0.3040
Domestic cat	0.6231	0.9662	0.3547
Drill	0.6883	0.9870	0.4544
Pig	0.7494	0.9029	0.6081

3 Correcting observed generation times.

Using the model we can estimate what the per generation rate and per year of a species would have been if we looked at a trio where the ages of the parents match the average generation time of the species. Using the fitted model we can calculate the rate per generation for species s using the scaling factor for that species ($x[s]$) and a average generation time instead of an observed age_mix:

$$\text{rate\_per\_generation}_s = (5.646 \times 10^{-9} + b \times \text{average\_generation\_time}_s) \times x[s] \tag{5}$$

And we can calculate the rate per year as:

$$\text{rate\_per\_year}_s = \frac{(5.6457625 \times 10^{-9} + b \times \text{average\_generation\_time}_s) \times x[s]}{\text{average\_generation\_time}_s} \tag{6}$$

This figure show the estimated rates (per year and generation) for trios where the age of the parents match the average generation time of the species:

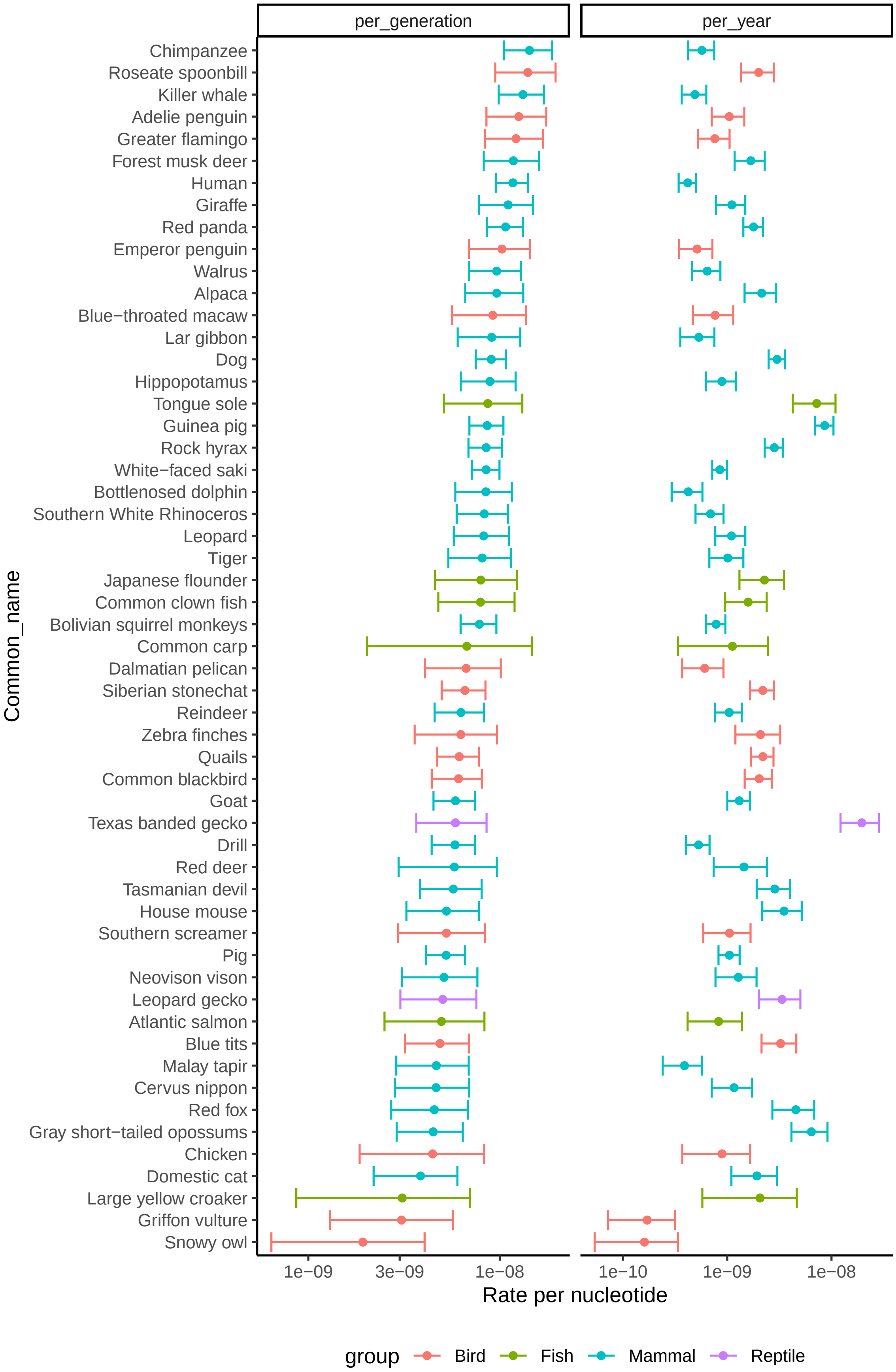

Figure 2: Estimated rate per genration and rate per year for each species

The large intercept that we observe in the models means that changes in the average generation time of a species can have a very large impact on the yearly mutation rate:

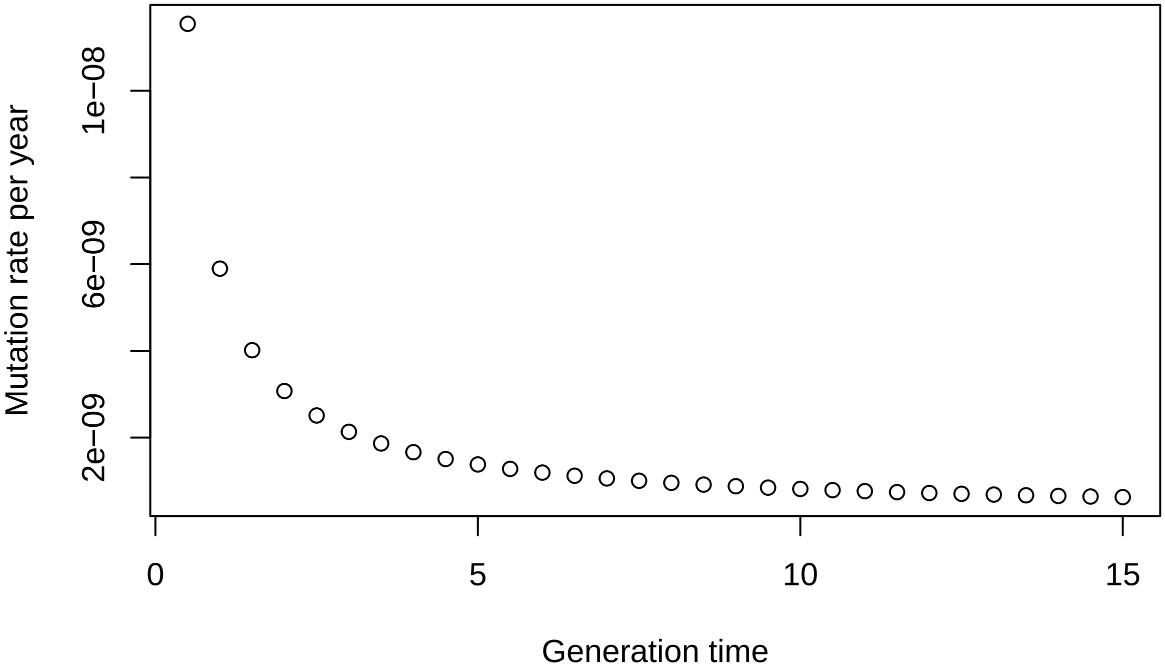

Figure 3: The effect of generation time on the rate per year

4 Validating model choice

4.1 Checking assumptions on parameters

In the model we use the exponential function as prior for both the intercept (a) and the slope (b) of the models. This means that we assume that the intercept and the slope cannot be negative. That is a fair assumption for the slope since it is impossible to remove mutations once they have occurred and thus there cannot be a negative correlation between the number of mutations and generation time. With regards to the intercept we cannot be as sure about the validity of the assumption. One could imagine a scenario where the parents didn't start accumulating mutations in their germ cells until they had reached a certain age, which would correspond to a model with a negative intercept. However in this data we see a clearly positive intercept, which we can also show by fitting a model without such assumptions. To make a model without assuming that the intercept is positive we have to use a log link function to ensure that λ is non-negative:

$$\begin{aligned} n_i &\sim \text{Poisson}(\lambda_i) \\ log(\lambda_i) &= \frac{\text{denominator}_i}{10^9} \times (a + b \times \text{age\_mix}_i)) \\ a &\sim \text{Norm}(0, 1) \\ b &\sim \text{Norm}(0, 1). \end{aligned} \tag{7}$$

The estimated parameters show that both a and b are clearly positive:

Table 5: Coefficients of log-link model

coef	mean	sd	2.5%	97.5%
a	0.815105563	0.0090234955	0.797412775	0.832531100
b	0.006967423	0.0006153289	0.005748733	0.008164415

But the use of the link function makes the values harder to interpret. We thus prefer the model with the exponential prior.

4.2 Model selection

The chosen model uses the same intercept for all species. While we do not have sufficient data to learn a separate intercept for each species it is possible that we could improve the model fit by having different intercepts for different groups of species. To test that we fitted a model with a different intercept for each of the four major classes of vertebrates:

$$\begin{aligned} n_i &\sim \text{Poisson}(\lambda_i) \\ \lambda_i &= \text{denominator}_i \times (a[group] + b \times \text{age\_mix}_i) \\ a[group] &\sim \text{Exp}(1) \\ b &\sim \text{Exp}(1). \end{aligned} \tag{8}$$

The results show that we end up with very similar estimates in each group:

Table 6: Coefficients of model with group intercept

coef	mean	sd	2.5%	97.5%
a[Bird]	5.237e-09	3.87e-10	4.505e-09	6.022e-09
a[Fish]	5.932e-09	8.32e-10	4.404e-09	7.644e-09
a[Mammal]	5.754e-09	2.35e-10	5.291e-09	6.218e-09
a[Reptile]	5.381e-09	9.81e-10	3.592e-09	7.429e-09
b	2.520e-10	2.20e-11	2.080e-10	2.960e-10

We can also estimate a model with a different slope for each group:

$$\begin{aligned} n_i &\sim \text{Poisson}(\lambda_i) \\ \lambda_i &= \text{denominator}_i \times (a + b[group] \times \text{age\_mix}_i) \\ a &\sim \text{Exp}(1) \\ b[group] &\sim \text{Exp}(1). \end{aligned} \tag{9}$$

The results show that we get an estimate in fish that is a bit higher than the other species but the credible intervals (highest posterior density intervals) overlap.

Table 7: Coefficients of model with group slope

coef	mean	sd	2.5%	97.5%
a	5.611e-09	2.14e-10	5.194e-09	6.028e-09
b[Bird]	2.340e-10	3.70e-11	1.630e-10	3.080e-10
b[Fish]	3.880e-10	2.30e-10	3.100e-11	9.030e-10
b[Mammal]	2.610e-10	2.50e-11	2.130e-10	3.100e-10
b[Reptile]	2.640e-10	1.99e-10	1.100e-11	7.430e-10

If we compare the model with a single intercept and single slope to the models with group specific intercepts of group specific slopes we see that the slightly better fit of the latter models do not warrent the added number of parameters:

Table 8: Comparison of different models

	WAIC	SE	dWAIC	dSE	pWAIC	weight
single_a_and_b	717.821	36.869	0.000	NA	4.120	0.906
group_a	723.159	36.370	5.338	4.096	7.969	0.063
group_b	724.534	37.342	6.713	3.279	8.489	0.032

Where WAIC is the "Widely Applicable Information Criteria" (*Wanatabe, Journal of Machine Learning Research, 2010*).
